# Knowledge and Use of Bee Products in Lithuania

**DOI:** 10.3390/nu17243927

**Published:** 2025-12-15

**Authors:** Juozas Labokas, Gintarė Kleibaitė

**Affiliations:** 1 Pharmacy and Pharmacology Center, Institute of Biomedical Sciences, Faculty of Medicine, Vilnius University, Geležinio Vilko g. 29A, LT-01112 Vilnius, Lithuania; kleibaite12@gmail.com; 2 Laboratory of Economic Botany, State Scientific Research Institute Nature Research Centre, Akademijos g. 2, LT-08412 Vilnius, Lithuania

**Keywords:** consumption, factors influencing use, nutritional knowledge, online survey, purposes of use, respondents, sociodemographic characteristics

## Abstract

**Background/Objective**: In recent years, there has been a growing public interest in natural products, including those derived from bees. While most scientific research on bee products has focused on their pharmacological properties, insufficient attention has been given to consumer knowledge, consumption habits and attitudes. The aim of this study was to estimate the popularity of use of different bee products and assess consumer knowledge about them in Lithuania. **Methods**: An online survey was carried out of the general adult population of Lithuania with 421 respondents included. **Results**: The study revealed that honey, beeswax and royal jelly were the best-known bee products, while bee venom was the least known one. Knowledge levels varied by age and occupation of respondents—older people and those working in pharmacy, healthcare, cosmetology, agriculture, beekeeping and food production showed better perception. Honey was most often used for treating colds (78.9%), prevention (78.1%) and reducing fever (65.3%). Dietary use of honey depended on demographic factors and was generally low—28.3% consumed it only a few times per year or less. For cosmetics, propolis was the most used product (34.2%). **Conclusions**: Older individuals and professionals in pharmacy, healthcare, cosmetology, agriculture, beekeeping and food production, demonstrated better knowledge of bee products. Although honey was rarely consumed as part of the diet, older people tended to use it more often than younger individuals. Men were more likely to use honey for treating digestive and circulatory issues and as an ingredient in food and beverages, whereas women were more inclined to use it for respiratory illnesses and cosmetic treatments.

## 1. Introduction

In recent years, there has been a growing public interest in natural products, including those derived from bees, or more exactly, Western honeybees (*Apis mellifera* Linnaeus, 1758). These insects provide not only honey as their name suggests, but an amazing diversity of other products and services. For example, Münstedt (2022) [1], classifies all bee products and services into six classes based on their specific origin and the ways people approach them in terms of utilization. However, the so-called classical or main bee products include only honey, propolis, royal jelly, bee bread, pollen, bee venom and beeswax [2].

While most scientific research on bee products has focused on their pharmacological properties [3,4,5,6], insufficient attention has been given to consumer knowledge, consumption habits and attitudes. The literature review has also shown that the use of bee products in various social groups is relatively poorly studied and depends on the geographical location of the country, traditions and other factors. These studies most often use survey and interview methods, preparing and distributing questionnaires and then analyzing the collected responses. One such study, conducted in Poland with 487 respondents showed that honey was mainly used for culinary purposes and somewhat less often for medical and cosmetic purposes. The other bee products were used much less often than honey: mainly beeswax and royal jelly for cosmetic purposes and propolis and bee pollen for medical purposes. It was also reported that the use of honey for different purposes depended on several factors, such as gender, age, income level and level of nutritional knowledge of the consumers as well as their self-assessed health status [7]. A study conducted in Slovakia, where 526 respondents were interviewed, simply aimed at mapping the situation with consumption of bee products and consumer preferences and showed that most Slovak consumers mainly consumed honey. Other bee products, such as propolis, royal jelly, bee pollen, bee bread or apilarnil, were consumed only occasionally or not at all [8]. A survey of 821 respondents in Hungary aimed at developing a strategy for Hungarian honey sales based on consumers’ purchase habits and found that out of the many types of honey produced in the country, people consumed only a few of them, indicating possibly a lack of knowledge. No significant differences were found between demographic groups, but it was revealed that the most important criteria when purchasing honey were quality, price, type of honey and quality of packaging [9]. A study conducted in Morocco, which surveyed 642 respondents, focused on understanding people’s preferences in terms of consistency, type, packaging, frequency of consumption, brands and purchasing criteria based on honey consumer profiles and revealed that honey consumption was influenced by perceptions of its medicinal uses, traditional staple foods and religion, attitudes towards beekeeping (better to buy honey from the market rather than online), honey consistency (preference for creamy and liquid honey), packaging (preference for glass containers), socio-demographic characteristics (gender, age, place of residence, income and level of education) and knowledge about the nutritional value of the bee product [10]. The review of foreign sources from the literature revealed that although studies on the use of bee products have slightly different objectives in every country, there are certain commonalities in methodological approaches such as mapping the consumption of bee products and consumer preferences as well as factors conditioning their behavior or habits.

A few studies on bee product use carried out in Lithuania focused either on a certain consumer group, like students [11], on a single municipality-level geographic area [12], on the demand for food supplements containing bee products [13] or on the quality assessment of different kinds of honey [14] with no attention paid to consumers’ knowledge of or motivation to use bee products.

According to the Lithuanian State Food and Veterinary Service, there were 11,230 registered apiaries with a total number of 196,479 bee colonies in Lithuania in 2019 [15]. The country’s richest ethnographic region by these beekeeping indicators is Aukštaitija [16]. However, beekeeping in general is a very old tradition in Lithuania, which is still popular across the country and can be considered to be a part of the national cultural heritage [17]. Consequently, the use of at least some bee products, such as honey and beeswax, has deep roots and is well-based on their traditional uses, let alone the latest scientific reports on them. Meanwhile, the use of other bee products may require more attempts by consumers to acquire reliable information on their properties and applications. Take as an example bee pollen, the most important components of which are full-fledged proteins (26–40%), amino acid content, which exceeds that of beef and eggs by five to seven times, and vegetable oil which amounts to 20% [18]. Similarly, bee bread, the stored form of bee pollen in the colony, has a high probiotic value and a higher yield than normal bee pollen and is another new health-oriented product [2]. In general, there are numerous reports about the use and biological activities of bee products showing they are rich in biologically active substances, vitamins, minerals and antioxidants, which is why they are used in both traditional and alternative medicine [19,20,21]. In this context, the role of consumers’ knowledge and their behavior or habits towards the use of bee products is of critical value. Moreover, one of the most acute problems of food safety, public health and veterinary medicine is the excessive use of antibiotics. By using some bee products for disease treatment or prevention, it would be possible to avoid synthetic antibiotics or at least reduce their dosage. Bee products may also be an alternative for treating antibiotic-resistant strains [18].

Based on the above-mentioned research gaps, the aim of this study was to estimate the popularity and motivation of use of different bee products and to assess consumers’ self-reported knowledge about them in Lithuania. The relationships between the habits of bee products use and sociodemographic factors were also investigated. Referring to the old tradition of beekeeping in Lithuania we expected that honey and beeswax should be the best known and most used bee products in the country. This survey-based research allowed us not only to gain a general understanding of bee product usage at a country-wide level but also suggested some implications for the future.

## 2. Materials and Methods

### 2.1. Questionnaire

The questionnaire for this survey-based research targeting the general Lithuanian adult population was prepared by employing Google Forms. It contained thirty-one questions including the request for confirmation for adult age and agreement to participate in the survey, eight questions regarding the sociodemographic characteristics of respondents, twenty questions addressing the frequency of use of bee products and knowledge about them and two final questions concerning self-assessment of health status and nutritional knowledge of respondents. Types of questions included sociodemographic questions, matrix type, open-ended, closed-ended, multiple-choice, as well as Likert scale [22]. The selection of survey questions was intended to collect comprehensive data on use habits and basic knowledge of bee products and was made after consulting relevant sources from the literature [8,9,23] (Table S1). The conceptual approach we used emphasized a logical rather than strictly mathematical understanding of the survey items [24]. Before conducting the main study, a questionnaire test was conducted on 4–11 April 2024. The electronic questionnaire was submitted to two Vilnius University students and two randomly selected individuals with no professional connection to medicine, the food industry or the cosmetics sector. After receiving their comments on unclear questions and wording, a respondent selection question was included and certain questions were adjusted, thus improving the clarity and validity of the questionnaire. The responses from the pilot study were not included in the analysis, and respondents who did not respond to the selection question were also excluded. The link to the online questionnaire was shared to Facebook groups and posted on websites, such as the Panevėžys Beekeepers’ Society, the Lithuanian Beekeepers’ Union, “Farmer’s Advisor” and the Faculty of Medicine of Vilnius University, from July to November 2024. To attract as many respondents as possible, participants were asked to share the survey link to increase the number of people who received the invitation to the survey and thus increase the number of participants in the survey. Participation in the survey was completely voluntary and anonymous.

### 2.2. Sample Size

Sample size for the survey was determined with an online sample size calculator [25] for the total Lithuanian adult population size of 2,395,290 [26]. Thus, it was established that the sample size should be 385 respondents to meet a 95% confidence interval and a 5% margin of error.

### 2.3. Statistical Analysis

Frequency tables were used to understand distribution patterns of responses and to find out which values were most common. The Shapiro–Wilk test was used to check the normality of the data distribution. Spearman’s correlation was employed to search for relationships between variables. Kruskal–Wallis H and Mann–Whitney U tests were used for multiple comparisons between groups [27]. The effect size for Mann–Whitney U test results was calculated with Microsoft 365 Excel Version 2511 by using the formula r = z/√N, where z is the standardized test statistic and N is the total sample size. The online calculators, based on Lenhard, W. and Lenhard, A. (2022) were also employed as provided in [28]. For classifying effect sizes, we referred to Cohen, 1992, where r values between 0.10 and 0.29, 0.30 and 0.49, and 0.50 or greater stand for small, medium and large effects sizes, respectively [29]. The Chi-square test (χ^2^) was used for categorical data to assess whether there were statistically significant differences between groups. Bonferroni correction was applied to reduce the probability of a type I error when performing multiple comparisons. Responses to open-ended questions were analyzed grouping responses by themes and counting frequencies. A 5% confidence level was used as default for statistical tests throughout the work.

The IBM SPSS Statistics (Version 30.0.0.0) predictive analytics software was used for data processing.

### 2.4. Sociodemographic Characteristics of Respondents

The study included data from 421 Lithuanian residents. To facilitate data comparison and simplify the assessment of differences between respondent groups, we regrouped the respondents regarding the categories of education, social status and occupation to make only 3 groups instead of 6–7 ones in each of these categories (see the original questionnaire in Table S1). Then, the sociodemographic characteristics of the respondents were summarized (Table 1).

As seen from Table 1, the distribution of respondents by gender showed that the majority were women—78.9% (n = 332). The largest share of participants belonged to the two age groups: 18–30 years old—39.2% (n = 165) and the 31–45 years old—35.4% (n = 149). Most respondents had higher education—68.6% (n = 289). Also, most respondents, 80.3% (n = 338), resided in urban areas. Regarding ethnographic regions, almost half of the participants, 47.5% (n = 200), lived in Aukštaitija, which is the largest of the five ethnographic regions of Lithuania. Most respondents were employed—66.7% (n = 281). The distribution of respondents by occupation revealed that most of them represented occupations other than those listed in the questionnaire (see Table S1)—52.7% (n = 222). More than half of respondents had an income of more than 1000 euros per month—55.6% (n = 234).

Thus, the picture of a median respondent of the survey was as follows: a young or middle-aged woman with higher education, urban resident of Aukštaitija, earning more than a thousand euros per month and occupied in a field other than pharmacy, healthcare, cosmetology, agriculture, beekeeping or food production. From our personal experience we know that this group of people are one of the most proactive in our society and who, inter alia, use the Internet as an everyday tool. They often take leading roles not only in local communities but also in families. Thus, the interpretation of the survey results should be treated bearing in mind this characteristic of the study sample.

## 3. Results and Discussion

### 3.1. Respondents’ Knowledge About Bee Products and Use Preferences

#### 3.1.1. Knowledge About Bee Products

**Figure 1:**
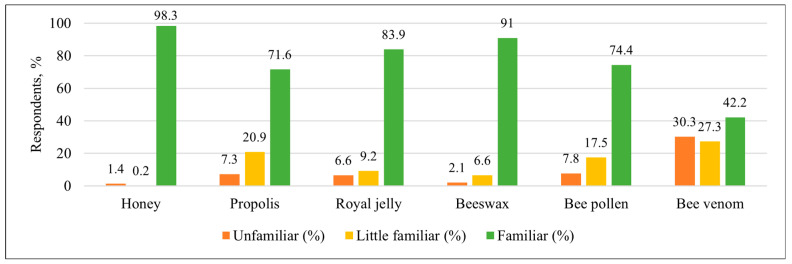
Distribution of respondents (%) by knowledge about bee products.

As expected, the results of this study showed that most of the respondents were familiar, or at least a little familiar, with honey. High percentages of familiarity were also observed for beeswax (91%, n = 383), royal jelly (83.9%, n = 353), bee pollen (74.4%, n = 313) and propolis (71.6%, n = 301). Meanwhile, bee venom received the least percentage of familiarity among respondents (42.2%, n = 178) (Figure 1).

Similar results were reported from other countries, e. g., in both Lithuania and Hungary, honey is the best-known bee product, but significantly less information is available about bee venom. In Poland, a different distribution trend was observed: although honey is also widely known, propolis and royal jelly are much less known than in Lithuania [7,9]. Differences from Polish population in knowledge of the mentioned bee products could be attributed to the educational differences in the respondents, as 68.6% of Lithuanian respondents had higher education vs. 60.6% of those in the Poland study.

In addition to the six most well-known bee products (Figure 1), more than a quarter of respondents (n = 116) indicated that they were aware of other bee products. Among them bee bread stood out as the most frequently mentioned product, receiving 100 mentions (i.e., 23.8% of all respondents) with drone brood homogenate (1.9%), beeswax moth products (0.7%), bee podmore (0.7%) and honeycomb (0.5%) receiving minor percentages of mentions.

**Figure 2:**
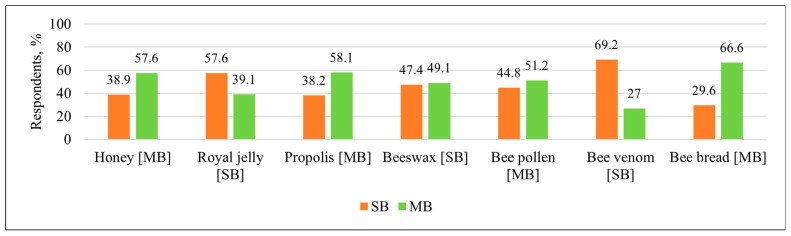
Distribution of respondents (%) by knowledge about bee products, answering the question of whether it is a product chemically synthesized by bees (SB) or modified by bees (MB). The abbreviations of the correct answers are given in square brackets next to the name of each product (Questionnaire clause: “If you don’t know, skip this question”).

The respondents’ answers showed that their knowledge on the origin of bee products differed significantly. The clearest attributions were observed for bee venom, with 295 respondents (69.2%) stating correctly that it is SB—synthesized by bees, and for bee bread, with 281 respondents (66.6%) stating correctly that it is MB—modified by bees. Honey was classified correctly as MB by 245 respondents (57.6%). Very similar percentages were obtained with royal jelly and propolis. The lowest percentages of correct answers were observed with beeswax (47.4%, n = 203, SB—correct) and bee pollen (51.2%, n = 220, MB—correct) (Figure 2).

**Figure 3:**
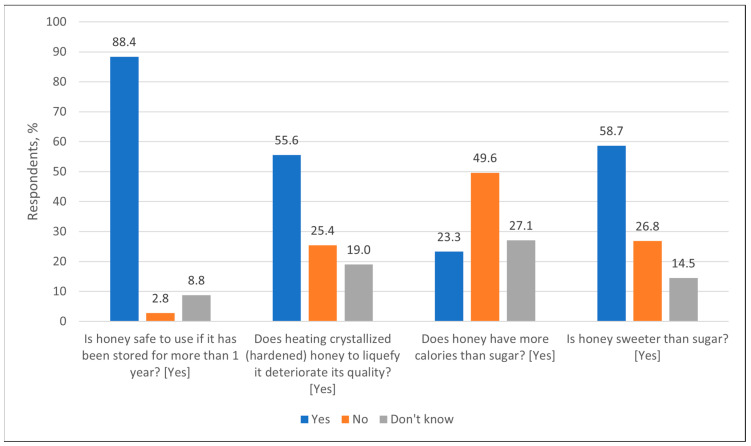
Distribution of respondents (%) by knowledge about properties of honey, answering the corresponding questions. Correct answers are given in square brackets next to each question.

Regarding knowledge of the properties of honey, most respondents answered correctly that honey is suitable for use after a year of storage (88.4%, n = 372) (Figure 3). Similar data were reported from other countries. For example, Polish consumers also mostly agree that honey can be stored for a long time and be suitable for consumption [7]. According to Eshete and Eshete (2019) [30], heating of crystallized honey deteriorates its quality because high temperatures destroy enzymes and vitamins, which in turn change the taste, color and other properties of honey. In our study, more than half of respondents (55.6%, n = 234) agreed that heating degrades the quality of honey. Similar data were reported from Slovakia, where consumers were aware that heating changes the properties of honey, but were not sufficiently informed about the loss of enzymes and vitamins [8]. A study conducted in Hungary showed that some consumers considered that heating has no effect on the quality of honey [9], which confirms that the lack of knowledge exists not only in Lithuania. Regarding the Lithuanian population we suppose that their knowledge on honey properties, particularly such practical knowledge as the effect of heating on its quality, spreads rapidly among regular consumers and they prefer buying honey just at the end of the honey harvesting season when it is not only cheaper but also more aromatic [31]. A recommendation even exists that high-quality honey, if purchased in winter or late autumn, should not be liquid. Otherwise, it may be last year’s honey that was heated to liquify it, a process during which the nutritional value of the product degrades significantly [32]. In our study, when asked about the calorie content of honey compared to sugar, 49.6% (n = 209) of respondents incorrectly stated that honey does not have more calories, 23.3% (n = 98) stated correctly that it does and 27.1% (n = 114) answered that they did not know. Similar responses were reported from Poland, where 57.8% of consumers incorrectly assessed the caloric content of honey, believing it to be lower in calories than sugar [7]. In Morocco, this misconception was even more widespread, with 64.7% of respondents believing that honey had fewer calories than sugar [10]. According to Samarghandian, honey is approximately 25% sweeter than sucrose due to its high fructose content [33]. Regarding the sweetness of honey, most respondents (58.7%, n = 247) stated correctly that honey is sweeter than sugar, while 26.8% (n = 113) disagreed with it and 14.5% (n = 61) did not know. Similar information was reported from other countries—the sweetness of honey was recognized by 47.5% of Moroccan [10], 52.8% of Slovak [8] and 60% of Polish respondents [7]. These results indicate that most respondents correctly understand the suitability of honey for storage, the effect of heating on quality and comparative sweetness, but there are still misconceptions about the calorie content of honey. In general, we assume that consumers’ knowledge of bee products depends on how interested they are in particular products and on what their personal preferences are.

#### 3.1.2. Bee Product Use Preferences

**Figure 4:**
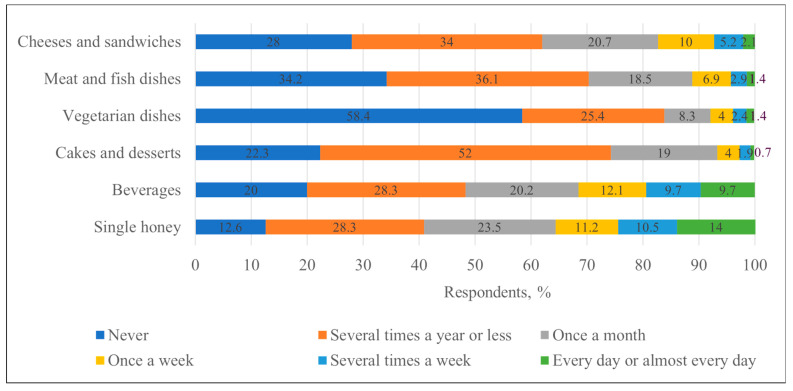
Distribution of respondents (%) by frequency of honey consumption with food, with beverages and as a single product.

Honey alone was most often consumed several times a year or less (28.3%, n = 119) or once a month (23.5%, n = 99). Beverages with honey were also most often consumed several times a year or less (28.3%, n = 119). Cakes and desserts with honey were rarely consumed, with 52.0% (n = 219) of respondents used to eating them several times a year or less. Vegetarian dishes with honey were the least popular—58.4% (n = 246) of respondents indicated that they never consumed them. Meat and fish dishes with honey were most often consumed several times a year or less (36.1%, n = 152), but 34.2% (n = 144) never consumed them. Cheese and sandwiches with honey were also a rare choice, with 34.0% (n = 143) of respondents used to eating them several times a year or less. In summary, most respondents used honey for food infrequently, usually a few times a year or less, and in some categories (e.g., vegetarian dishes), most respondents indicated that they did not use it at all (Figure 4).

Studies conducted in Poland found that residents there consumed honey or honey products more frequently than the participants in the current study, with the most common response being once a month [7] or even several times per month in some regions [34]. The latter source refers to health benefits, a wide range of culinary uses, flavor and habits as the main incentives for honey use. And it could also be applied to Lithuanian consumers that for the increase in honey consumption nutritional education is needed. In Morocco, more than 64% of respondents consumed honey only occasionally and only 12.7% included it in their meals [10].

**Figure 5:**
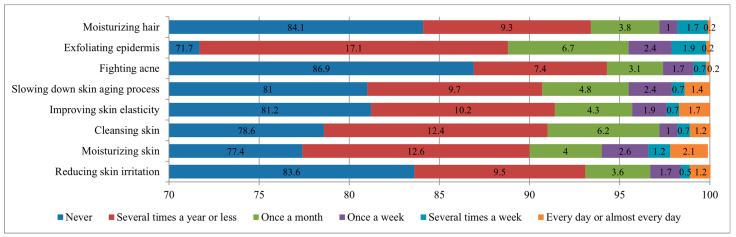
Distribution of respondents (%) by frequency of honey use for cosmetic purposes.

When analyzing the use of honey for cosmetic purposes, it turned out that most respondents used it rarely or not at all. The largest number of respondents (70–85%) indicated that they never used honey for cosmetic purposes. A similar situation was reported from Poland, where more than 65% of consumers did not use this product for cosmetic purposes, and from Morocco, where this indicator exceeded 70% [7,10]. It was most often used for exfoliation (28.3% of respondents at least once a year) and for skin hydration (22.6% of respondents). Other uses, such as reducing skin irritation, improving elasticity, or slowing down the aging process, were even less common, with only 10–20% of respondents choosing them at least several times a year (Figure 5).

Comparable data were reported from Poland, where honey was also used for the same cosmetic purposes, and most often used for skin moisturizing (23%), reducing irritation (19.1%), exfoliating the epidermis (18.2%) and improving skin elasticity (17.7%) [7]. These indicators could be attributed to some cultural similarities between the two countries.

**Figure 6:**
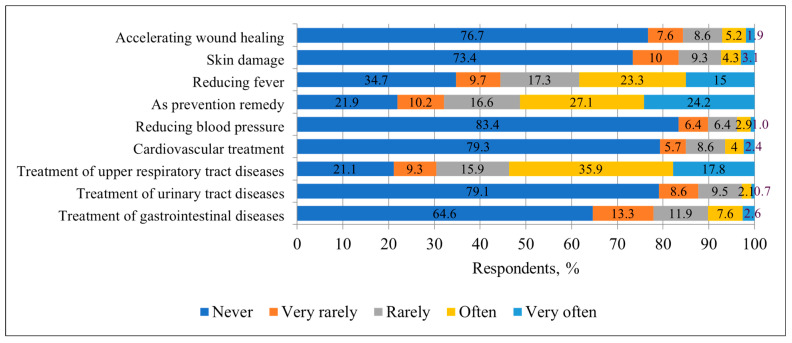
Distribution of respondents (%) by frequency of honey use for healthcare purposes.

When analyzing the use of honey for therapeutic purposes, it turned out that most respondents did not use it as a therapeutic agent. Most often, people used honey for the treatment of upper respiratory tract diseases—as many as 78.9%. Also, some respondents used it for illness prevention (78.1%) and fever reduction (65.3%). This revealed that although honey is considered beneficial for health, its therapeutic application is limited mainly to colds, prevention of illness and fever reduction (Figure 6).

Compared to surveys conducted in other countries, the use of honey for therapeutic purposes was also reported from Poland and Morocco. A similar survey conducted in Poland found that 42.2% of respondents used honey for prevention of illness, 33.6% for treating colds and 28.7% for reducing fever [7]. In Morocco, 43.38% of respondents used honey for prevention and only 10.36% for reducing fever [10]. This indicates that honey was used for therapeutic purposes more frequently in Lithuania than in Poland and Morocco. This could be related to the higher educational level of Lithuanian respondents (68.6% with higher education), compared to Polish (60.6%) and Moroccan (63%) respondents as well as to different cultural traditions regarding the African country.

**Figure 7:**
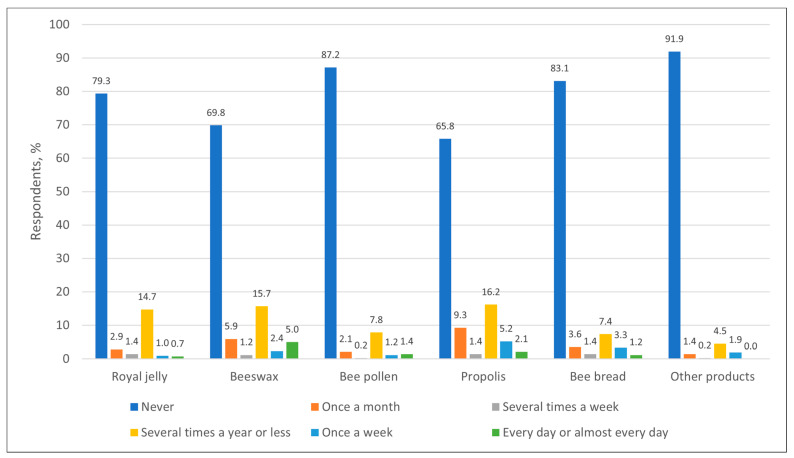
Distribution of respondents (%) by frequency of use of bee products (other than honey) for cosmetics.

Analyzing the frequency of use of bee products for cosmetics, it could be seen that most respondents did not use them regularly. Royal jelly (n = 334), pollen (n = 367) and bee bread (n = 350) were never used by more than 79% of respondents. Beeswax and propolis were used at least occasionally for cosmetic purposes by more people, but regular use (once a week or more often) was extremely low. Propolis was the most popular product used for cosmetics—34.2% (n = 132) of respondents used it at least once a year. But in general, this shows that bee products are rarely used for cosmetic purposes (Figure 7).

**Figure 8:**
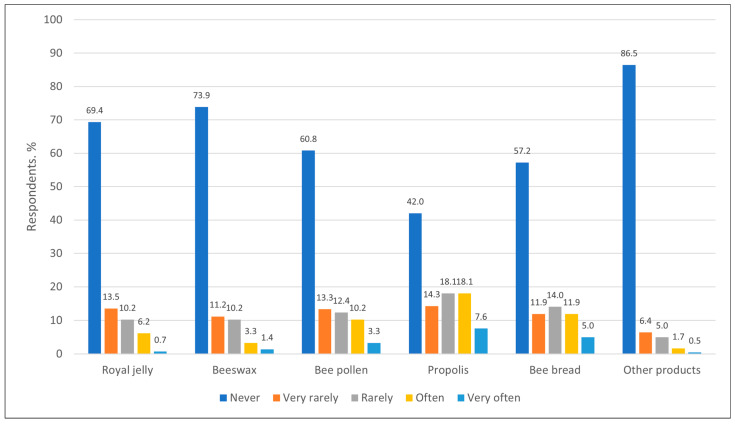
Distribution of respondents (%) by frequency of use of bee products (other than honey) for healthcare purposes.

Regarding the use of bee products for healthcare purposes, the most used one was propolis, which was used often or very often by 25.6% (n = 108) of respondents. Bee pollen and bee bread were also used more often than other products—they were rarely or often used by 23.3% (n = 98) and 25.9% (n = 109) of respondents, respectively. Other bee products were almost never used for healthcare purposes, i.e., more than 86% (n = 364) of respondents did not use them (Figure 8).

Comparable data was reported from Poland, where propolis was also the most popular product used for healthcare purposes, with 25.5% of respondents using it. However, bee pollen was used less frequently there than in Lithuania, with only 13% of respondents using it. Other bee products, such as beeswax or royal jelly, were used in medicine even less frequently [7]. Interestingly, in 2023, Lithuania was among the top five countries in the world by honey consumption with 1.90 kg/person, while Poland with its 0.83 kg/person ranked 29th [35].

#### 3.1.3. Factors Determining Use of Bee Products

**Figure 9:**
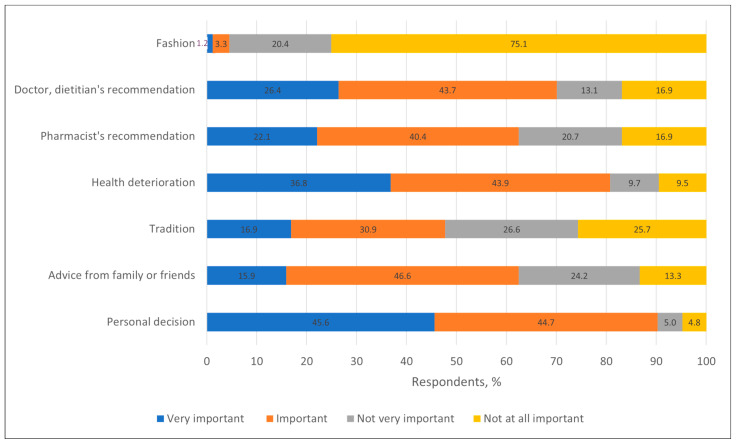
Distribution of respondents (%) by factors determining their use of bee products.

The use of bee products by most respondents was determined by personal decision—192 (45.6%) indicated it as very important and 188 (44.7%) as important. Deterioration of health was also a significant factor—155 (36.8%) considered it very important and 185 (43.9%) as important. Recommendations of dietitians were considered important by 184 (43.7%) and very important by 111 (26.4%), and recommendations of pharmacists were considered important by 170 (40.4%) and very important by 93 (22.1%) of respondents. Compared to the study conducted in Poland, the opinion of healthcare professionals is more important in Lithuania (3). Advice from family or friends was important for 196 (46.6%) respondents and very important for 67 (15.9%) respondents, and tradition was important for 130 (30.9%) respondents and very important for 71 (16.9%) respondents. For Fashion, in terms of considering the use of bee products fashionable, was identified as completely unimportant by 316 (75.1%) respondents (Figure 9). This suggests that respondents are unbiased regarding social desirability, as most bee products are considered natural and healthy by society.

**Figure 10:**
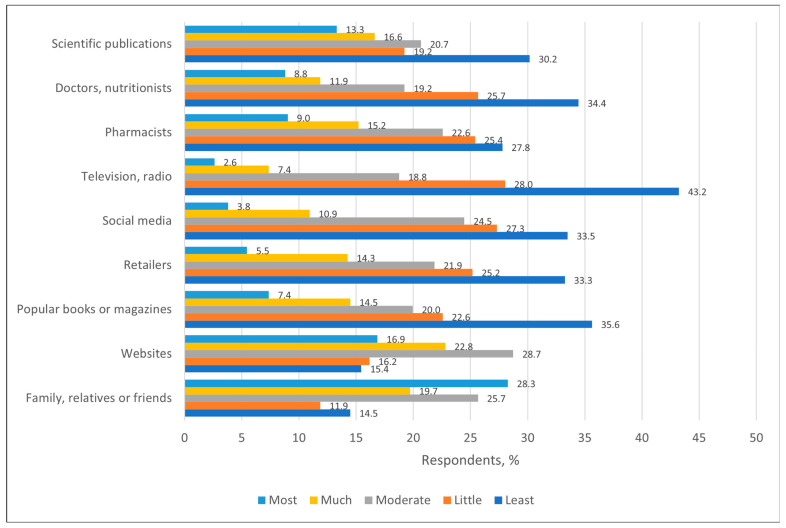
Distribution of respondents (%) by their sources of information about bee products.

Regarding the ways of obtaining information about bee products, the most popular ones were family, relatives or friends (28.3%, n = 119) and websites (16.9%, n = 71). The least popular source of information was television and radio (43.2%, n = 182) (Figure 10).

When compared with a similar survey conducted in Poland, the trends appeared similar. In Poland, the greatest influence on information about bee products is exerted by family and friends (32.5%), as well as the Internet (28.7%) [7]. Studies conducted abroad confirm that the Internet and social networks, as in many other cases, are becoming an increasingly important source of information about bee products, especially among younger consumers [10].

**Figure 11:**
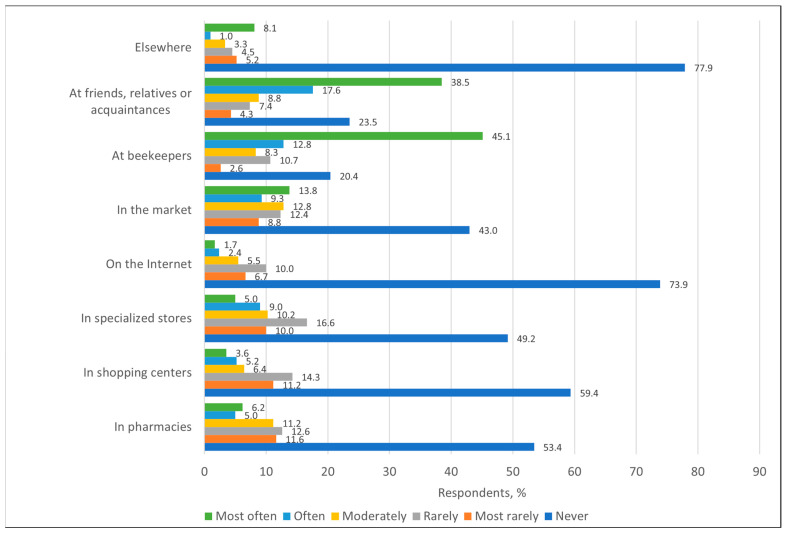
Distribution of respondents (%) by their places of purchase of bee products.

When summarizing information on places where bee products were purchased, the results of the survey showed that bee products were most often purchased from beekeepers (n = 190, 45.0%) and from friends, relatives or acquaintances (n = 162, 38.4%). Bee products were least often purchased online (n = 7, 1.7%) and in supermarkets (n = 15, 3.6%). As many as 73.7% (n = 311) of respondents never bought bee products online and 59.2% (n = 250) in supermarkets (Figure 11).

Similar trends were observed in other countries, with studies showing that consumers tend to choose bee products based on authenticity and trust in the seller, with beekeepers being seen as more reliable suppliers than supermarkets [10]. In Poland, most consumers buy honey from beekeepers (45.53%) [7]. In Serbia, there is also a trend to buy honey directly from manufacturers (44.3% of respondents) [36].

The results of the survey showed that for most respondents it is important that the bee products they purchase are of local origin, i.e., Lithuanian. A percentage of 80.8% (n = 341) of respondents indicated that this is very important, while only 15.9% (n = 67) indicated that it is not very important to them. Similar trends were observed in other countries. In Romania, local honey is particularly valued due to distrust of imported products [9]. In Hungary, 70% of consumers emphasize the value of local honey [9]. Studies conducted abroad also show that consumers most often choose local bee products due to their authenticity and support for local beekeepers [8].

Respondents’ attitudes towards the type of honey by botanical origin were varied: 33.9% (n = 143) indicated that it is very important to them, while 34.6% (n = 146) stated that it is not very important to them. One fifth, or 19.9% (n = 84), of respondents noted that it is important for them that it is nectar honey, not honeydew, while 11.4% (n = 48) answered that the type of honey does not matter to them at all. Of the respondents for whom the botanical origin of honey is very important (n = 143, 33.9%), the largest proportion prefers linden honey (n = 86, 60.1%) which complies with the findings from Poland [37]. Interestingly, a recent study carried out in Spain suggests that the imagination of the honey production landscape can have a real impact on the choice of the botanical origin of honey [38]. Buckwheat honey is chosen by 18 respondents (12.6%), while dandelion honey is indicated by 11 respondents (7.7%). Raspberry honey was chosen by seven respondents (4.9%) and heather honey by six respondents (4.2%). Meanwhile, it has been reported that buckwheat and heather honey, i.e., dark honey types, often have higher contents of minerals and other compounds compared to linden, raspberry or other light honey types. Moreover, honeydew is considered superior to pure honey in terms of food and medicinal remedy [39]. Thus, the respective choices of respondents can be attributed to their weak knowledge of chemical properties or simply to the preferences regarding product color, smell or storage behavior. It has been reported that globally there is some differentiation in the demand for different types of honey and in many countries nectar honey is valued more highly than honeydew honey but, in other countries, honeydew honey is preferred [40]. According to our data Lithuania belongs to the former group.

Respondents were also asked whether they agreed with the statement that bee products should not be used therapeutically in cases where the chemical justification is unknown. Most respondents (n = 308, 73.2%) agreed with this statement.

Of the 100 respondents who listed side effects of bee products, allergic reactions were the most common, reported by 80% of participants. Skin reactions were experienced by 8%, digestive disorders by 7%, changes in blood sugar levels by 2% and other effects accounted for 3%.

Respondents were asked how they assessed their health. The majority (n = 208, 49.3%) rated their health as “Good”, while 113 respondents (26.8%) rated it as “Very good” and 93 participants (22.0%) rated their health as “Intermediate” between good and bad. Only a small proportion of respondents rated their health as “Bad” (n = 4, 0.9%) or “Very bad” (n = 3, 0.7%).

Finally, participants were asked about their level of nutritional knowledge. Most respondents rated their knowledge as “Intermediate” between low and high (n = 209, 49.5%), while 172 respondents (40.8%) rated it as “High”, and 28 respondents (6.6%) rated their level of nutritional knowledge as “Very high”. A small proportion of participants rated their knowledge as “Low” (n = 10, 2.4%) or “Very low” (n = 2, 0.5%).

### 3.2. Relationships of Knowledge and Use of Bee Products with Sociodemographic Characteristics

#### 3.2.1. Relationships with Age

To determine whether the age group of respondents has a significant impact on the knowledge about bee products, a Kruskal–Wallis H test was performed. The results showed that there were statistically significant differences in the knowledge of four bee products: honey (*p* = 0.018), propolis (*p* < 0.001), bee pollen (*p* = 0.002) and bee venom (*p* = 0.001) (Table 2).

To determine which age groups differed significantly, a Mann–Whitney U test was performed. Bonferroni correction was applied separately for each bee product, evaluating six pairwise comparisons between age groups, resulting in an adjusted significance level of *p* < 0.0083. The 31–45 age group had better knowledge of bee venom (*p* < 0.001) and bee pollen (*p* = 0.003) than the 18–30 age group. The 46–60 age group had significantly better knowledge of propolis (*p* = 0.026), bee pollen (*p* = 0.004) and bee venom (*p* = 0.001) than the 18–30 age group; however, the significance of bee venom knowledge did not persist after Bonferroni correction. The 61+ age group had better knowledge of bee venom (*p* = 0.003), but less knowledge of honey (*p* = 0.005), compared to the 18–30 age group. Compared to the 31–45 age group, the 61+ age group also had better knowledge of bee venom (*p* = 0.035), but this result was not significant after Bonferroni correction. Spearman’s rank correlation test revealed that age significantly and positively, although weakly, correlated with the variety of known bee products (ρ = 0.236, *p* < 0.05), i.e., a weak tendency could be observed that the number of known bee products increases along with age (Table 3).

The effect size (r) values following significant differences showed small effects with r varying from 0.141 (difference in knowledge of propolis between the age groups 18–30/46–60) to 0.219 (difference in knowledge of propolis between the age groups 18–30/31–45 (Table 3). Nevertheless, this suggests that customer age is a meaningful factor in knowledge of bee products.

Using Spearman’s rank correlation test, it was found that age correlated with honey consumption in food statistically significantly and positively (ρ = 0.274, *p* < 0.05). Therefore, it can be stated that there is a weak tendency that with increasing age, honey consumption in food also increases. A comparable result was reported from Poland, where honey was consumed more frequently by people aged over 46 years [23]. One possible reason for that, particularly in older adults, is that when people age, their sensory functions, including taste and smell, generally decline, which affects their food choices [41].

The Kruskal–Wallis H test was used to determine whether age group had a significant effect on the frequency of honey consumption in various food and beverage products. The results showed a statistically significant effect of age group in three of the six product categories: consumption of honey alone (*p* < 0.001), consumption of vegetarian dishes with honey (*p* < 0.001) and consumption of cheeses and sandwiches with honey (*p* < 0.001) differed between different age groups (Table 4).

In these three product categories, the Mann–Whitney U test revealed which age groups had statistically significant differences. Younger people (18–30 years old) consumed honey significantly less frequently in all three categories than older age groups (31–61+ years). The frequency of single honey consumption significantly differed between all age groups—older people consumed it more often than younger people. All age groups consumed honey in vegetarian dishes and cheeses, sandwiches with honey statistically significantly more often than the 18–30 years age group (Table 5).

The effect size (r) values following significant differences showed a medium effect size for age group in frequency of honey use with vegetarian dishes, with r = 0.306 (between age groups 31–45/61+), r = 0.421 (between 18 and 30/61+) and r = 0.453 (between 18 and 30/46–60). A medium effect size was also established in frequency of honey use with cheeses and sandwiches between the 18–30 and 46–60 age groups (r = 0.329). The rest of the significant differences showed small effect sizes but mostly meaningful (Table 5). One plausible explanation of relatively large effect sizes could be the fact that these foods are considered healthy ones, while aging people need such products more than young ones.

The Kruskal–Wallis H test was also performed to determine whether age groups have a significant impact on the frequency of honey use for various cosmetic purposes. The results showed statistically significant differences between age groups in three categories: the use of honey for improving skin elasticity (*p* = 0.005), slowing down skin aging process (*p* = 0.002) and exfoliating epidermis (*p* = 0.004) (Table 6).

The Mann–Whitney U test revealed statistically significant differences between most age groups. Respondents older than 61 years of age used honey significantly more often to improve skin elasticity compared to younger age groups. To slow down the skin aging process, respondents 61 years of age and older used honey more often than those of the 31–45 and 18–30 age groups. Meanwhile, the youngest respondents (18–30 years) used honey significantly more often for epidermal exfoliation than representatives of the 46–60 and 61+ age groups. Age statistically significantly and positively correlated with the use of honey for cosmetics (ρ = 0.118, *p* < 0.05). The use of honey for cosmetics also increases with age (Table 7).

As seen from Table 7, the largest effect size (r = 0.293), which is close to medium, was obtained in difference in frequency of honey use for improving skin elasticity between age groups 46–60/61+, and next to it was slowing down skin aging between the youngest and the oldest age groups, 18–30/61+ (r = 0.275). The effect size of the difference in honey use for exfoliating epidermis between the latter two groups was a bit smaller (r = 0.225) but still meaningful. This is understandable as skin condition is one of the major indicators of an organism’s aging and of the general health status of a person to some degree. Therefore, the use of natural products for skin care becomes increasingly relevant along the aging process. It has been reported that honey represents one of the most reported ingredients of animal origin in cosmetic applications [42].

The Kruskal–Wallis H test was performed to determine whether different age groups differed significantly in the frequency of honey use for healthcare purposes. The results showed that age groups differed statistically significantly in most uses of honey for healthcare (Table 8).

Therefore, an additional test, the Mann–Whitney U test, was performed to determine specific differences between age groups. Statistically significant differences between age groups were found in several categories related to the use of honey for healthcare purposes. Older age groups (46–60 years and 61+ years) used honey for health problems significantly more often than younger groups (18–30 years and 31–45 years). Age statistically significantly and positively correlated with the use of honey for healthcare purposes (ρ = 0.187; *p* < 0.05). Compared to the 18–30 years age group, individuals aged 61+ years were more likely to use honey for digestive problems (*p* < 0.001) with a medium effect size (r = 0.355), cardiovascular disorders (*p* < 0.001) with a medium effect size (r = 0.366), blood pressure regulation (*p* < 0.001) with a small effect size (r = 0.286), skin lesions (*p* < 0.001) with a medium effect size (r = 0.356) and wound healing (*p* < 0.001) with a medium effect size (r = 0.336). Compared to the 31–45 years group, individuals aged 61+ years more often used honey to solve cardiovascular problems (*p* < 0.001), medium effect size (r = 0.307); reduce fever (*p* = 0.007), small effect size (r = 0.205); treat gastrointestinal diseases (*p* < 0.001), small effect size (r = 0.271); reduce blood pressure (*p* = 0.003), small effect size (r = 0.227); treat skin lesions (*p* < 0.001), medium effect size (r = 0.298); and accelerate wound healing (*p* = 0.001), small effect size (r = 0.248) (Table 9).

In general, that could be explained by the fact that people’s interest in natural or alternative ways of healthcare increases with age. Also, the authors’ personal observations may confirm that old people, particularly in the countryside, tend to use more natural products, like honey, herbal teas, etc., to replenish and maintain their health condition.

The Kruskal–Wallis H test was performed to determine whether different age groups differ significantly in the frequency of use of bee products as cosmetics. The results showed statistically significant differences between age groups in the frequency of use of bee pollen (*p* < 0.001) and bee bread (*p* = 0.048) (Table 10).

The Mann–Whitney U test identified several statistically significant differences between age groups related to the use of bee products for cosmetics. Respondents of 61+ years of age used bee propolis for cosmetic purposes significantly more often than those aged 18–30 and 31–45 years with a medium effect size (r = 0.302). Similarly, the 61+ age group used bee bread for cosmetics statistically significantly more often than the 18–30 age group, although with a small effect size (r = 0.203) (Table 11).

The results of the Kruskal–Wallis H test showed that the use of bee products for healthcare purposes differed statistically significantly between age groups (*p* < 0.001 or *p* = 0.001) (Table 12).

The results of the Mann–Whitney U test showed that the use of bee products for healthcare purposes differed significantly between age groups. The youngest group (18–30 years old) used most bee products less frequently than the older groups, and the largest differences were observed when compared it with the oldest (61+ years) age group (*p* < 0.001; *p* = 0.002). Significantly less frequently used in the 18–30 years group were royal jelly (*p* = 0.003 compared to 31–45 years, *p* < 0.001 compared to 46–60 years and *p* = 0.002 compared to 61+ years group), with a small effect size (r varied from 0.168 to 0.225), beeswax (*p* < 0.001 compared to 61+ years, r = 0.280), bee pollen (*p* < 0.001 compared to all groups, r = 0.248 ÷ 0.367), propolis (*p* < 0.001 compared to all groups, r = 0.222 ÷ 0.396), bee bread (*p* < 0.001 compared to all groups, r = 0.213 ÷ 0.353) and other bee products (*p* < 0.001 compared to the oldest group, r = 0.385). Significant differences were also seen in the use of propolis between the 61+ years group and the 31–45 years (*p* < 0.001) and 46–60 years (*p* = 0.003) groups, with effect sizes r = 0.257 and 0.284. The use of bee bread was more common in the 61+ years group than in the 31–45 years group (*p* = 0.005, r = 0.212), and other bee products were also used significantly more often in the oldest group than in the 31–45 age group (*p* < 0.001, r = 0.366) and 46–60 age group (*p* = 0.001, r = 0.313) (Table 13). In general, an evident increase in effect size from small to medium was observed along with age.

#### 3.2.2. Relationships with Gender

The Mann–Whitney U test revealed that the use of honey for food and beverages, cosmetics and factors determining its use differed statistically significantly between men and women. Women used honey more often for cosmetic procedures (*p* = 0.002), while men used honey alone or as an ingredient in food and beverages (*p* = 0.019). Although the small effect sizes in both cases (r = 0.149 and 0.115, respectively) (Table 14) do not allow for any deeper inferences to be made, such a trend may be attributed to the culturally developed behavior of the local population. Since only single comparisons were made between the two gender groups for each investigated category separately, the Bonferroni correction was not applied.

Men were statistically significantly more likely than women to use honey to treat gastrointestinal diseases (*p* = 0.009), to lower blood pressure (*p* = 0.017) and to accelerate wound healing (*p* = 0.032). Meanwhile, women were significantly more likely than men to use honey to treat upper respiratory diseases (*p* = 0.001). However, the effect sizes of all these differences were small (r = 0.075 to 0.158) (Table 15).

#### 3.2.3. Relationships with Occupation

The Kruskal–Wallis H test showed that the knowledge of bee products, knowledge of the properties of honey, the use of honey in food, the use of honey for cosmetics, and the use of honey for healthcare purposes had *p*-values less than 0.05 (Table 16). Therefore, in these cases, the Mann–Whitney U test was performed to determine between which groups the mean ranks differed statistically significantly.

The Mann–Whitney U test showed that the mean ranks were statistically significantly different between the occupational groups “Other” and “Pharmacy, Healthcare, Cosmetology” (*p* = 0.002) and between “Other” and “Agriculture, Beekeeping, Food production” (*p* = 0.001). It is understandable that individuals representing other occupations have less knowledge about bee products than those working in the fields of pharmacy, healthcare, cosmetology, agriculture, beekeeping or food production. The analysis also revealed that representatives of the occupation group “Agriculture, Beekeeping and Food production” were statistically significantly more likely to use honey for cosmetics than those who indicated the occupation “Other” (*p* = 0.011). It was also found that representatives of the occupation group “Agriculture, Beekeeping and Food production” were significantly more likely to use honey for healthcare purposes compared to the group “Other” (*p* = 0.013). The largest effect sizes considering significant differences were observed between occupation groups Other and Agriculture, Beekeeping, Food production in four categories (r = 0.388 to 0.571) and between Other and Pharmacy, Healthcare, Cosmetology in the category Knowledge of bee products (r = 0.306) (Table 17). This was expected as professionals in pharmacy, healthcare, agriculture or food production are more exposed to the subject of study.

The Kruskal–Wallis H test revealed statistically significant differences in the use of royal jelly (*p* < 0.001), bee pollen (*p* = 0.004), bee propolis (*p* < 0.001) and bee bread (*p* = 0.008) for healthcare purposes, comparing different occupational groups (Table 18).

The Mann–Whitney U test showed that the use of bee products for healthcare purposes differed statistically significantly between occupational groups (*p* < 0.0167). The representatives of the “Agriculture, Beekeeping and Food production” occupational group used propolis more often (*p* < 0.001) than those of the “Pharmacy, Healthcare and Cosmetology” group. Meanwhile, specialists of the “Pharmacy, Healthcare and Cosmetology” group used royal jelly more often (*p* < 0.001) than those of the occupation group “Other”. It was also found that the “Agriculture, Beekeeping and Food production” group used bee pollen (*p* = 0.001), propolis (*p* < 0.001) and bee bread (*p* = 0.002) more often than the group “Other”. Regarding effect sizes of significant differences, the occupational groups had small effects (r = 0.158 to 0.284) (Table 19).

#### 3.2.4. Relationships with Social Status

The Kruskal–Wallis H test showed that the knowledge of bee products, the knowledge of properties of honey and its use for food differed statistically significantly between occupational groups (*p* < 0.05) (Table 20). Therefore, the Mann–Whitney U test was performed to determine which groups had statistically significant differences in mean ranks.

The Mann–Whitney U test showed that the knowledge of bee products differed statistically significantly by the social status. The mean ranks of the “Students” and “Employed” groups were significantly different (*p* = 0.001), suggesting that employed respondents had more knowledge about bee products than students. However, the analysis revealed that employed respondents know statistically significantly less about the properties of honey compared to students (*p* = 0.008). It was also found that employed respondents used honey as an ingredient in food or drinks significantly more often compared to students (*p* = 0.01). The effect size estimation of the significant differences revealed relatively large effect sizes between Student and Employed groups in all three categories (r = 0.510 to 0.514) (Table 21).

#### 3.2.5. Relationships with Education

The results of the Kruskal–Wallis H test showed that the use of royal jelly (*p* = 0.003) and beeswax (*p* = 0.029) for healthcare purposes differed significantly between respondents by education (Table 22).

The Mann–Whitney U test showed that individuals with higher education were statistically significantly more likely to use royal jelly (*p* < 0.001) and beeswax (*p* = 0.015) for healthcare purposes compared to individuals with secondary, specialized secondary or advanced vocational education and training. However, effect sizes in both cases were low (r = 0.167 and 0.120, respectively) (Table 23).

## 4. Conclusions

Analysis of the study results based on responses from 421 adult individuals, suggests that bee products may be classified into three groups by knowledge to the population of Lithuania, such as, the most widely known products (honey, beeswax and royal jelly), less known products (bee pollen, propolis) and least known products (bee venom and others). In general, the knowledge of bee products depends on age, occupation and social status. The variety of known bee products correlates positively with the age of respondents. And this could be partly attributed to the fact that people generally take more care regarding their health as they age by appreciating the potential of natural products. Moderate effect sizes (r = 0.306 ÷ 0.492) of statistically significant differences in knowledge between specialists of pharmacy, healthcare, cosmetology, agriculture, beekeeping and food production vs. other occupations suggest that there is a significant gap in knowledge about bee products and help to identify target groups of the Lithuanian population requiring more education. The fact that employed individuals are better informed about bee products than students could be interpreted as employed people generally being older and having more experience than students. In general, a significant part of the society studied still exhibits misconceptions about the origin and properties of some bee products.

Bee products in Lithuania are most often used for healthcare purposes, such as prevention of illnesses and easing respiratory problems. For cosmetic purposes, bee products are used less often than for healthcare, with propolis standing out as the most used bee product both for healthcare and cosmetic purposes. Men use honey more often to treat digestive and circulatory problems and as an ingredient in food and beverages, while women prioritize it for respiratory diseases and cosmetic procedures. We assume that these differences have some gender-related cultural background as it is commonly accepted that men seek to be physically strong while women mostly focus on their appearance. In nutrition, honey is used relatively rarely—usually only a few times a year or less, but older people choose it more often than younger people. With increasing age, the use of honey and bee propolis for cosmetic purposes also increases. In general, specialists in agriculture, beekeeping and food production use bee products more often than representatives of other fields. Education is an important factor in conditioning the use of bee products as it correlates positively with honey use for healthcare purposes.

Apart from personal choice and health condition, specialist recommendations are one of the key factors influencing the use of bee products. Among the most important sources of information on bee products are family, friends and the Internet. Local, i.e., Lithuanian, bee products are more appreciated than imported ones and are most often purchased directly from beekeepers. This could be considered as a certain kind of tie strengthening local societies. Although priorities regarding the botanical origin of honey differ, linden honey is the most highly valued one, but buckwheat, dandelion, raspberry and heather honeys are also in demand.

Although there are some limitations to this study, it implies that the dissemination of qualified information about bee products and their use, as well as targeted consumer education to increase their knowledge, is of the highest priority to maximize benefits from this kind of natural products. To achieve this, the respective educational programs should be established and the existing ones strengthened.

## 5. Limitations

This survey was conducted by using an online questionnaire which has a certain disadvantage if compared to the live interview method. For example, the interpretation of some questions may be too subjective, which is not an issue in live interviews. This should be considered in future research, possibly by combining online surveys with components of live or computer-assisted web interviews. Another possible limitation is related to dissemination of the survey link through Facebook groups, beekeeping organizations and a university website which may have introduced some selection bias of respondents. To more fully assess the knowledge of the Lithuanian population about bee products and their consumption preferences, especially among the older population of the country, who usually do not use the Internet, it is recommended to conduct ethnopharmacological field studies covering different ethnographic regions of Lithuania. This is important also regarding the selection of an unbiased sample of the population, because the current study showed that the median respondent was a young or middle-aged woman with higher education, urban resident, etc., i.e., a group of people who are proactive in everyday life including frequent use of the Internet. Such studies would not only allow for a more detailed understanding of consumption traditions and related cultural characteristics in different regions but would also provide a more generalized picture of the situation and better background for new scientific research on bee products.

Also, the representatives of such sectors as food production, pharmacies, medicine and cosmetology should be addressed separately to obtain more specialized data on the role of bee products in their respective areas.

## Figures and Tables

**Table 1:** Sociodemographic characteristics of respondents after regrouping data (see text above).

Sociodemographic Category	Value of Sociodemographic Category	Number of Respondents (N)	Percentage of Respondents (%)
Gender	Female	332	78.9
	Male	86	20.4
	Other	3	0.7
Age group (yrs.)	18–30	165	39.2
	31–45	149	35.4
	46–60	83	19.7
	≥61	24	5.7
Education	Primary, Basic and Other	13	3.1
	Secondary, Special secondary and Advanced vocational education and training	119	28.3
	Higher	289	68.6
Residence	Rural area	83	19.7
	Urban area	338	80.3
Ethnographic region	Aukštaitija	200	47.5
	Dzūkija	52	12.4
	Mažoji Lietuva	47	11.2
	Suvalkija	31	7.4
	Žemaitija	91	21.6
Social status	Employed	281	66.5
	Student	91	21.6
	Unemployed	49	11.9
Occupation	Pharmacy, Healthcare, Cosmetology	156	37.1
	Agriculture, Beekeeping, Food production	43	10.2
	Other	222	52.7
Income, Eur/month	<500	41	9.7
	>1000	234	55.6
	500–1000	85	20.2
	Undisclosed	61	14.5

**Table 2:** Significance of differences in knowledge of bee products between age groups (Kruskal–Wallis H test).

Bee Product	H Statistic	df	*p*-Value
Honey	10.092	3	0.018 *
Propolis	17.843	3	<0.001 *
Royal jelly	2.925	3	0.403
Beeswax	0.951	3	0.813
Bee pollen	14.454	3	0.002 *
Bee venom	15.574	3	0.001 *

* Indicates statistically significant differences; df—degrees of freedom.

**Table 3:** Significance of differences in knowledge of four bee products between age groups (Mann–Whitney U test).

Age Groups	Total	Honey			Propolis			Bee Pollen			Bee Venom		
Pairwise	N	Z-Value	*p*-Value	r	Z-Value	*p*-Value	r	Z-Value	*p*-Value	r	Z-Value	*p*-Value	r
18–30/31–45	314	−0.072	0.942	0.004	−3.888	<0.001 **	0.219	−2.981	0.003 **	0.168	−1.466	0.143	0.083
18–30/46–60	248	−1.224	0.221	0.078	−2.226	0.026 *	0.141	−2.891	0.004 **	0.184	−3.208	0.001 **	0.204
18–30/61+	189	−2.823	0.005 **	0.205	−1.669	0.095	0.121	−1.819	0.069	0.132	−2.934	0.003 **	0.213
31–45/46–60	232	−1.21	0.262	0.079	−1.094	0.274	0.072	−0.445	0.656	0.029	−1.858	0.063	0.122
31–45/61+	173	−2.661	0.008 **	0.202	−0.027	0.978	0.002	−0.413	0.68	0.031	−2.111	0.035 *	0.160
46–60/61+	107	−1.341	0.180	0.130	−0.540	0.589	0.052	−0.141	0.888	0.014	−1.005	0.315	0.097

* Indicates statistically significant differences; ** indicates significance after Bonferroni correction (*p* < 0.0083); r—effect size.

**Table 4:** Significance of differences in frequency of honey use in various food product forms between age groups (Kruskal–Wallis H test).

Product Form	H Statistic	df	*p*-Value
Single honey	77.185	3	<0.001 *
Beverages	4.304	3	0.230
Cakes and desserts	3.790	3	0.285
Vegetarian dishes	17.628	3	<0.001 *
Meat and fish dishes	2.471	3	0.481
Cheeses and sandwiches	37.712	3	<0.001 *

* Indicates statistically significant differences; df—degrees of freedom.

**Table 5:** Significance of differences in frequency of honey use in three food product forms between age groups (Mann–Whitney U test).

Age Groups	Total	Single Honey			Vegetarian Dishes with Honey			Cheeses and Sandwiches with Honey		
Pairwise	N	Z-Value	*p*-Value	r	Z-Value	*p*-Value	r	Z-Value	*p*-Value	r
18–30/31–45	314	−5.062	<0.001 **	0.286	−3.899	<0.001 **	0.220	−3.536	<0.001 **	0.200
18–30/46–60	248	−7.131	<0.001 **	0.453	−2.534	0.011 **	0.161	−5.177	<0.001 **	0.329
18–30/61+	189	−5.781	<0.001 **	0.421	−2.5	0.012 **	0.182	−3.787	<0.001 **	0.275
31–45/46–60	232	−3.302	<0.001 **	0.217	−0.621	0.535	0.041	−2.697	0.007 **	0.177
31–45/61+	173	−4.024	0.0260 *	0.306	−0.637	0.379	0.048	−2.448	0.382	0.186
46–60/61+	107	−2.222	<0.001 **	0.215	−0.879	0.524	0.085	−0.875	0.014 *	0.085

* Indicates statistically significant differences (*p* < 0.05); ** indicates significance after Bonferroni correction (*p* < 0.0083); r—effect size.

**Table 6:** Significance of differences in frequency of honey use for cosmetic purposes between age groups (Kruskal–Wallis H test).

Purpose of Honey Use	H Statistic	df	*p*-Value
Reducing skin irritation	6.034	3	0.110
Moisturizing skin	4.640	3	0.200
Cleansing skin	7.462	3	0.059
Improving skin elasticity	12.702	3	0.005 *
Slowing down skin aging process	15.032	3	0.002 *
Fighting acne	0.410	3	0.938
Exfoliating epidermis	13.275	3	0.004 *
Moisturizing hair	2.952	3	0.399

* Indicates statistically significant differences; df—degrees of freedom.

**Table 7:** Significance of differences in frequency of honey use for three cosmetic purposes between age groups (Mann–Whitney U test).

Age Groups	Total	Improving Skin Elasticity			Slowing Down Skin Aging			Exfoliating Epidermis		
Pairwise	N	Z-Value	*p*-Value	r	Z-Value	*p*-Value	r	Z-Value	*p*-Value	r
18–30/31–45	314	−0.719	0.472	0.041	−0.765	0.444	0.043	−1.986	0.047 *	0.112
18–30/46–60	248	−0.602	0.547	0.038	−1.701	0.089	0.108	−2.652	0.008 **	0.168
18–30/61+	189	−2.897	0.004 **	0.211	−3.783	<0.001 **	0.275	−3.093	0.002 **	0.225
31–45/46–60	232	−0.011	0.991	0.001	−0.998	0.319	0.066	−0.950	0.342	0.062
31–45/61+	173	−3.424	<0.001 **	0.260	−3.165	0.002 **	0.241	−1.993	0.046 *	0.152
46–60/61+	107	−3.032	0.002 **	0.293	−2.212	0.027 *	0.214	−1.470	0.142	0.142

* Indicates statistically significant differences; ** indicates significance after Bonferroni correction (*p* < 0.0083); r—effect size.

**Table 8:** Significance of differences in frequency of honey use for healthcare purposes between age groups (Kruskal–Wallis H test).

Purpose of Honey Use	H Statistic	df	*p*-Value
Treatment of gastrointestinal diseases	31.685	3	<0.001 *
Treatment of urinary tract diseases	5.797	3	0.122
Treatment of upper respiratory tract diseases	3.322	3	0.345
Treatment of cardiovascular disorders	30.227	3	<0.001 *
Reducing blood pressure	17.242	3	<0.001 *
Preventive remedy	9.542	3	0.023 *
Reducing fever	8.999	3	0.029 *
Treatment of skin lesions	27.746	3	<0.001 *
Acceleration of wound healing	23.427	3	<0.001 *

* Indicates statistically significant differences; df—degrees of freedom.

**Table 9:** Significance of differences in frequency of honey use for healthcare purposes between age groups (Mann–Whitney U test).

**Age Groups**	**Total**	**Treatment of Gastrointestinal Diseases**			**Treatment of Cardiovascular Disorders**			**Reducing Blood Pressure**			**Preventive Remedy**		
**Pairwise**	**N**	**Z**	***p***	**r**	**Z**	***p***	**r**	**Z**	***p***	**r**	**Z**	***p***	**r**
18–30/31–45	314	−2.058	0.032 *	0.116	−1.166	0.244	0.066	−1.207	0.227	0.068	−2.113	0.035 *	0.119
18–30/46–60	248	−4.12	<0.001 **	0.262	−3.499	<0.001 **	0.222	−2.588	0.010 *	0.164	−1.397	0.163	0.089
18–30/61+	189	−4.883	<0.001 **	0.355	−5.026	<0.001 **	0.366	−3.928	<0.001 **	0.286	−2.629	0.009 *	0.191
31–45/46–60	232	−2.423	0.015 *	0.159	−2.367	0.018 *	0.155	−1.504	0.133	0.099	−0.444	0.657	0.029
31–45/61+	173	−3.563	<0.001 **	0.271	−4.037	<0.001 **	0.307	−2.985	0.003 **	0.227	−1.568	0.117	0.119
46–60/61+	107	−1.631	0.103	0.158	−2.097	0.036 *	0.203	−1.634	0.102	0.158	−1.817	0.069	0.176
**Age Groups**		**Total**	**Reducing Fever**				**Treatment of Skin Lesions**			**Accelerating Wound Healing**			
**Pairwise**		**N**	**Z**	***p***	**r**		**Z**	***p***	**r**	**Z**	***p***		**r**
18–30/31–45		314	−1.466	0.143	0.083		−1.557	0.12	0.088	−1.958	0.05		0.110
18–30/46–60		248	−0.639	0.523	0.041		−3.206	0.001 **	0.204	−3.063	0.002 **		0.195
18–30/61+		189	−1.908	0.056	0.139		−4.893	<0.001 **	0.356	−4.613	<0.001 **		0.336
31–45/46–60		232	−1.889	0.059	0.124		−1.889	0.059	0.124	−1.336	0.182		0.088
31–45/61+		173	−2.693	0.007 **	0.205		−3.916	<0.001 **	0.298	−3.264	0.001 **		0.248
46–60/61+		107	−1.582	0.114	0.153		−2.296	0.022 *	0.222	−2.15	0.032 *		0.208

* Indicates statistically significant differences; ** indicates significance after Bonferroni correction (*p* < 0.0083); Z—standardized test statistic; *p*—significance level; r—effect size.

**Table 10:** Significance of differences in frequency of use of bee products for cosmetic purposes between age groups (Kruskal–Wallis H test).

Bee Product	H Statistic	df	*p*-Value
Royal jelly	3.824	3	0.281
Beeswax	1.304	3	0.728
Bee pollen	4.966	3	0.174
Propolis	20.598	3	<0.001 *
Bee bread	7.914	3	0.048 *
Other products (drone brood, bee venom)	2.650	3	0.449

* Indicates statistically significant differences; df—degrees of freedom.

**Table 11:** Significance of differences in frequency of use of two bee products for cosmetic purposes between age groups (Mann–Whitney U test).

Age Groups	Total	Propolis			Bee Bread		
Pairwise	N	Z-Value	*p*-Value	r	Z-Value	*p*-Value	r
18–30/31–45	314	−0.300	0.765	0.017	−1.004	0.315	0.057
18–30/46–60	248	−1.970	0.490 *	0.125	−1.319	0.187	0.084
18–30/61+	189	−4.155	<0.001 **	0.302	−2.786	0.005 **	0.203
31–45/46–60	232	−1.657	0.098	0.109	−0.416	0.677	0.027
31–45/61+	173	−3.975	<0.001 **	0.302	−2.073	0.038 *	0.158
46–60/61+	107	−2.772	0.006 **	0.268	−1.678	0.093	0.162

* Indicates statistically significant differences; ** indicates significance after Bonferroni correction (*p* < 0.0083); r—effect size.

**Table 12:** Significance of differences in frequency of use of bee products for healthcare purposes between age groups (Kruskal–Wallis H test).

Bee Product	H Statistic	df	*p*-Value
Royal jelly	18.033	3	<0.001 *
Beeswax	15.982	3	0.001 *
Bee pollen	35.700	3	<0.001 *
Propolis	39.685	3	<0.001 *
Bee bread	32.254	3	<0.001 *
Other products	31.350	3	<0.001 *

* Indicates statistically significant differences; df—degrees of freedom.

**Table 13:** Significance of differences in frequency of use of bee products for healthcare purposes between age groups (Mann–Whitney U test).

**Age Groups**	**Total**	**Royal Jelly**			**Beeswax**			**Bee Pollen**		
**Pairwise**	**N**	**Z**	***p***	**r**	**Z**	***p***	**r**	**Z**	***p***	**r**
18–30/31–45	314	−2.973	0.003 **	0.168	−1.525	0.127	0.086	−4.387	<0.001 **	0.248
18–30/46–60	248	−3.533	<0.001 **	0.224	−2.383	0.017 *	0.151	−4.064	<0.001 **	0.258
18–30/61+	189	−3.09	0.002 **	0.225	−3.845	<0.001 **	0.280	−5.039	<0.001 **	0.367
31–45/46–60	232	−1.057	0.29	0.069	−1.107	0.268	0.073	−0.322	0.748	0.021
31–45/61+	173	−1.431	0.153	0.109	−2.773	0.006 **	0.211	−2.518	0.012 *	0.191
46–60/61+	107	−0.618	0.537	0.060	−1.705	0.88	0.165	−2.118	0.034 *	0.205
**Age Groups**	**Total**	**Propolis**			**Bee Bread**			**Other Products**		
**Pairwise**	**N**	**Z**	***p***	**r**	**Z**	***p***	**r**	**Z**	***p***	**r**
18–30/31–45	314	−3.937	<0.001 **	0.222	−3.773	<0.001 **	0.213	−0.472	0.637	0.027
18–30/46–60	248	−3.911	<0.001 **	0.248	−3.831	<0.001 **	0.243	−1.794	0.073	0.114
18–30/61+	189	−5.449	<0.001 **	0.396	−4.855	<0.001 **	0.353	−5.293	<0.001 **	0.385
31–45/46–60	232	−0.527	0.598	0.035	−0.814	0.415	0.053	−1.345	0.179	0.088
31–45/61+	173	−3.379	<0.001 **	0.257	−2.783	0.005 **	0.212	−4.812	<0.001 **	0.366
46–60/61+	107	−2.937	0.003 **	0.284	−1.973	0.048 *	0.191	−3.242	0.001 **	0.313

* Indicates statistically significant differences (*p* < 0.05); ** indicates significance after Bonferroni correction (*p* < 0.0083); Z—standardized test statistic; *p*—significance level; r—effect size.

**Table 14:** Significance of differences in knowledge of bee products and in frequency of honey use between men and women (Mann–Whitney U test).

Category	Gender	N	Mean Rank	Z-Value	*p*-Value	r
Knowledge of bee products	Man	86	221.47	−1.063	0.288	0.052
	Woman	332	206.40			
Use of honey for food	Man	86	236.74	−2.352	0.019 *	0.115
	Woman	332	202.44			
Use of honey for cosmetics	Man	86	177.14	−3.052	0.002 *	0.149
	Woman	332	217.88			
Use of honey for healthcare	Man	86	204.15	−0.462	0.644	0.023
	Woman	332	210.89			

* Indicates statistically significant differences; N—sample size; r—effect size.

**Table 15:** Significance of differences in frequency of honey use for healthcare purposes between men and women (Mann–Whitney U test).

Purpose of Honey Use	Gender	N	Mean Rank	*p*-Value	z-Value	r
Treatment of gastrointestinal diseases	Man	86	235.46	0.009 *	−2.236	0.109
	Woman	332	202.78			
Treatment of urinary tract diseases	Man	86	215.25	0.481	−0.495	0.024
	Woman	332	208.01			
Treatment of upper respiratory tract diseases	Man	86	171.94	0.001 *	−3.235	0.158
	Woman	332	219.23			
Treatment of cardiovascular disorders	Man	86	220.96	0.16	−0.987	0.048
	Woman	332	206.53			
Lowering blood pressure	Man	86	227.22	0.017 *	−1.526	0.075
	Woman	332	204.91			
Preventive remedy	Man	86	206.05	0.761	−0.296	0.014
	Woman	332	210.39			
Reducing fever	Man	86	194.24	0.174	−1.313	0.064
	Woman	332	213.45			
Treatment of skin lesions	Man	86	220.01	0.242	−0.905	0.044
	Woman	332	206.78			
Accelerating wound healing	Man	86	227.74	0.032 *	−1.571	0.077
	Woman	332	204.77			

* Indicates statistically significant differences; N—sample size; r—effect size.

**Table 16:** Significance of differences in knowledge of bee products and in frequency of honey use between occupational groups (Kruskal–Wallis H test).

Category	Group by Occupation	N	Mean Rank	χ^2^	df	*p*-Value
Knowledge of bee products	Pharmacy, Healthcare, Cosmetology	156	229.36	23.611	2	<0.001 *
	Agriculture, Beekeeping, Food production	43	269.27			
	Other	222	186.81			
Knowledge of honey properties	Pharmacy, Healthcare, Cosmetology	156	223.64	7.381	2	0.025 *
	Agriculture, Beekeeping, Food production	43	239.08			
	Other	222	196.68			
Use of honey in food	Pharmacy, Healthcare, Cosmetology	156	194.70	20.068	2	<0.001 *
	Agriculture, Beekeeping, Food production	43	287.51			
	Other	222	207.63			
Use of honey for cosmetics	Pharmacy, Healthcare, Cosmetology	156	212.24	8.490	2	0.014 *
	Agriculture, Beekeeping, Food production	43	255.48			
	Other	222	201.52			
Use of honey for healthcare purposes	Pharmacy, Healthcare, Cosmetology	156	205.94	8.563	2	0.014 *
	Agriculture, Beekeeping, Food production	43	262.31			
	Other	222	204.62			

* Indicates statistically significant differences; N—sample size; χ^2^—Chi-square statistic; df—degrees of freedom.

**Table 17:** Significance of differences in knowledge of bee products and in frequency of honey use between occupational groups (Mann–Whitney U test).

Category	Groups by Occupation Pairwise	Difference Between Mean Ranks	*p*-Value	r
Knowledge of bee products	Other/Pharmacy, Healthcare, Cosmetology	−42.55	0.002 **	0.306
	Other/Agriculture, Beekeeping, Food production	−82.46	0.001 **	0.492
	Pharmacy, Healthcare, Cosmetology/Agriculture, Beekeeping, Food production	−39.91	0.148	0.121
Knowledge of honey properties	Other/Pharmacy, Healthcare, Cosmetology	−26.96	0.090	0.262
	Other/Agriculture, Beekeeping, Food production	−42.4	0.097	0.609
	Pharmacy, Healthcare, Cosmetology/Agriculture, Beekeeping, Food production	−15.44	1.000	0.154
Use of honey for food	Pharmacy, Healthcare, Cosmetology/Other	−12.93	0.924	0.198
	Pharmacy, Healthcare, Cosmetology/Agriculture, Beekeeping, Food production	−92.81	0.048 *	0.287
	Other/Agriculture, Beekeeping, Food production	−79.88	0.049 *	0.388
Use of honey for cosmetics	Other/Pharmacy, Healthcare, Cosmetology	−10.72	1.000	0.174
	Other/Agriculture, Beekeeping, Food production	−53.96	0.011 **	0.571
	Pharmacy, Healthcare, Cosmetology/Agriculture, Beekeeping, Food production	−43.24	0.072	0.005
Use of honey for healthcare	Other/Pharmacy, Healthcare, Cosmetology	−1.32	1.000	0.165
	Other/Agriculture, beekeeping, Food production	−57.69	0.013 **	0.532
	Pharmacy, Healthcare, Cosmetology/Agriculture, Beekeeping, Food production	−56.37	0.021 *	0.058

* Indicates statistically significant differences; ** indicates significance after Bonferroni correction (*p* < 0.0167); r—effect size.

**Table 18:** Significance of differences in frequency of use of bee products for healthcare purposes between occupational groups (Kruskal–Wallis H test).

Bee Product	H Statistic	df	*p*-Value
Royal jelly	15.376	2	<0.001 *
Beeswax	3.473	2	0.176
Bee pollen	10.952	2	0.004 *
Propolis	21.720	2	<0.001 *
Bee bread	9.584	2	0.008 *
Other products	0.392	2	0.822

* Indicates statistically significant differences; df—degrees of freedom.

**Table 19:** Significance of differences in frequency of use of bee products for healthcare purposes between occupational groups (Mann–Whitney U test).

Groups by Occupation	Total	Royal Jelly			Bee Pollen			Propolis			Bee Bread		
Pairwise	N	Z	*p*	r	Z	*p*	r	Z	*p*	r	Z	*p*	r
Pharmacy, Healthcare, Cosmetology/Agriculture, Beekeeping, Food production	199	−0.716	0.474	0.051	−2.222	0.026 *	0.158	−3.35	<0.001 **	0.237	−2.493	0.013 **	0.177
Pharmacy, Healthcare, Cosmetology/Other	378	−3.887	<0.001 **	0.200	−1.485	0.138	0.076	−1.823	0.68	0.094	−0.913	0.361	0.047
Agriculture, Beekeeping, Food production/Other	265	−1.754	0.079	0.108	−3.25	0.001 **	0.200	−4.616	<0.001 **	0.284	−3.042	0.002 **	0.187

* Indicates statistically significant differences; ** indicates significance after Bonferroni correction (*p* < 0.0167); Z—standardized test statistic; *p*—significance level; r—effect size.

**Table 20:** Significance of differences in knowledge of bee products, knowledge of honey properties and frequency of honey use between different social status groups (Kruskal–Wallis H test).

Category	Social Status	N	Mean Rank	χ^2^	df	*p*-Value
Knowledge of bee products	Employed	280	223.88	13.145	2	0.001 *
	Student	91	172.31			
	Unemployed	50	209.28			
Knowledge of honey properties	Employed	280	200.91	9.201	2	0.010 *
	Student	91	244.29			
	Unemployed	50	206.91			
Use of honey for food	Employed	280	219.99	9.258	2	0.010 *
	Student	91	176.77			
	Unemployed	50	222.97			
Use of honey for cosmetics	Employed	280	211.58	0.766	2	0.682
	Student	91	203.86			
	Unemployed	50	220.76			
Use of honey for healthcare	Employed	280	213.08	3.486	2	0.175
	Student	91	193.23			
	Unemployed	50	231.72			

* Indicates statistically significant differences; N—sample size; χ^2^—Chi-square statistic; df—degrees of freedom.

**Table 21:** Significance of differences in knowledge of bee products, knowledge of honey properties and frequency of honey use for food between different social status groups (Mann–Whitney U test).

Category	Social Status Pairwise	Difference Between Mean Ranks	*p*-Value	r
Knowledge of bee products	Student/Unemployed	−36.97	0.225	0.439
	Student/Employed	−51.57	0.001 *	0.510
	Unemployed/Employed	−14.60	1.000	0.526
Knowledge of honey properties	Employed/Unemployed	−6.00	1.000	0.525
	Employed/Student	−43.38	0.008 *	0.514
	Unemployed/Student	−37.38	0.223	0.438
Use of honey for food	Student/Employed	−43.22	0.010 *	0.510
	Student/Unemployed	−46.20	0.092	0.444
	Employed/Unemployed	−2.98	1.000	0.527

* Indicates statistically significant differences after Bonferroni correction (*p* < 0.0167); r—effect size.

**Table 22:** Significance of differences in frequency of use of bee products for healthcare purposes between groups by education (Kruskal–Wallis H test).

Bee Product	H Statistic	df	*p*-Value
Royal jelly	11.351	2	0.003 *
Beeswax	7.099	2	0.029 *
Bee pollen	5.711	2	0.058
Propolis	2.437	2	0.296
Bee bread	5.592	2	0.061
Other products	3.073	2	0.215

* Indicates statistically significant differences; df—degrees of freedom.

**Table 23:** Significance of differences in frequency of use of bee products for healthcare purposes between groups by education (Mann–Whitney U test).

Groups by Education Pairwise	Royal Jelly			Beeswax		
	Z-Value	*p*-Value	r	Z-Value	*p*-Value	r
Primary, Basic, Other/Secondary, Specialized secondary, Advanced vocational education and training	−1.305	0.192	0.114	−1.906	0.057	0.166
Primary, Basic, Other/Higher	−0.034	0.973	0.002	−0.852	0.394	0.049
Secondary, Specialized secondary, Advanced vocational education and training/Higher	−3.365	<0.001 *	0.167	−2.424	0.015 *	0.120

* Indicates statistically significant differences after Bonferroni correction (*p* < 0.0167); r—effect size.

## Data Availability

The original contributions presented in this study are included in the article. Further inquiries can be directed at the corresponding author.

## Supplementary Materials

The following supporting information can be downloaded at: https://www.mdpi.com/article/10.3390/nu17243927/s1, Table S1: Questionnaire form for “Knowledge and Use of Bee Products in Lithuania”.
