# Supplementary material for: Knowledge and Use of Bee Products in Lithuania
Source: Nutrients. 2025 Dec 15;17(24):3927. doi: 10.3390/nu17243927 (PMC12735761; doi:10.3390/nu17243927)
Supplement: Supplementary file 1 [file nutrients-17-03927-s001.zip › nutrients-4010162-supplementary.pdf]

Table S1

Questionnaire form for “Knowledge and Use of Bee Products in Lithuania“

1. Can you confirm that you are an adult and agree to participate in this survey?
   - Yes
   - No

General Part:

2. Your gender:
   - Male
   - Female
   - Other
3. Your age group (years):
   - 18–30
   - 31–45
   - 46–60
   - 61 and more
4. Your education:
   - Primary
   - Basic
   - Secondary
   - Advanced vocational education and training / Special secondary
   - Higher
   - Other
5. Your social status:
   - Student
   - Employed
   - Employed and student
   - Unemployed
   - Retired (not employed)
   - On maternity/parental leave
   - Other
6. Your occupation:
   - Related to agriculture
   - Beekeeping
   - Pharmacy
   - Healthcare
   - Cosmetology
   - Food production
   - Other
7. Your income, Eur/month:
   - <500
   - 500–1,000
   - >1,000
   - I do not wish to disclose
8. You are:
   - Urban resident
   - Rural resident
9. You reside in:
   - Aukštaitija
   - Dzūkija
   - Suvalkija
   - Žemaitija
   - Lithuania Minor

Special Part:

10. What bee products are you familiar with?

	Unfamiliar	Little familiar	Familiar
Honey	○	○	○
Royal jelly	○	○	○
Propolis	○	○	○
Beeswax	○	○	○
Bee pollen	○	○	○
Bee venom	○	○	○

11. What bee products do you know that were not included in the previous question? If you don't know, skip this question.

____

12. Which of these products are chemically synthesized by bees themselves (SB), and which are derived from plants and modified by bees (MB)? If you don't know, skip this question.

	SB	MB
Honey	○	○
Royal jelly	○	○
Propolis	○	○
Beeswax	○	○
Bee pollen	○	○
Bee venom	○	○
Bee bread	○	○

13. How much do you know about the properties of honey?

	Yes	No	Do not know
Is honey safe to use if it has been stored for more than 1 year?	○	○	○
Does heating crystallized (hardened) honey to liquefy it deteriorate its quality?	○	○	○
Does honey have more calories than sugar?	○	○	○
Is honey sweeter than sugar?	○	○	○

14. What is the difference between pure and raw honey? If you don't know, move on to the next question.

____

15. How often do you use honey (alone or as an ingredient) in food/beverages?

	Never	Several times a year or less	Once a month	Once a week	Several times a week	Every day or almost every day
Honey alone	○	○	○	○	○	○
Beverages	○	○	○	○	○	○
Cakes and desserts	○	○	○	○	○	○
Vegetarian dishes	○	○	○	○	○	○
Meat and fish dishes	○	○	○	○	○	○
Cheese and sandwiches	○	○	○	○	○	○

16. How often do you use honey as cosmetics for these purposes?

	Never	Several times a year or less	Once a month	Once a week	Several times a week	Every day or almost every day
To reduce skin irritation	○	○	○	○	○	○
For skin hydration	○	○	○	○	○	○
For skin cleansing	○	○	○	○	○	○
For improving skin elasticity	○	○	○	○	○	○
To slow down the skin aging process	○	○	○	○	○	○
To fight acne	○	○	○	○	○	○
For epidermal exfoliation	○	○	○	○	○	○
For hair hydration	○	○	○	○	○	○

17. How often do you use honey for healthcare purposes?

	Never	Very rarely	Rarely	Often	Very often
For the treatment of gastrointestinal diseases	○	○	○	○	○
For the treatment of urinary tract diseases	○	○	○	○	○
For the treatment of upper respiratory tract diseases	○	○	○	○	○
For the treatment of cardiovascular disorders	○	○	○	○	○
For reducing blood pressure	○	○	○	○	○
As preventive remedy	○	○	○	○	○
For reducing fever	○	○	○	○	○
For the treatment of skin lesions	○	○	○	○	○
For accelerating wound healing	○	○	○	○	○

18. How often do you use these bee products for cosmetics?

	Never	Several times a year or less	Once a month	Once a week	Several times a week	Every day or almost every day
Royal jelly	○	○	○	○	○	○
Beeswax	○	○	○	○	○	○
Bee pollen	○	○	○	○	○	○
Propolis	○	○	○	○	○	○
Bee bread	○	○	○	○	○	○
Other products (drone brood, bee venom)	○	○	○	○	○	○

19. How often do you use these bee products for healthcare purposes?

	Never	Very rarely	Rarely	Often	Very often
Royal jelly	○	○	○	○	○
Beeswax	○	○	○	○	○
Bee pollen	○	○	○	○	○
Propolis	○	○	○	○	○
Bee bread	○	○	○	○	○
Other (drone brood, bee venom)	○	○	○	○	○

20. What factors determine the use of bee products?

	It doesn't matter at all	Not very important	Important	Very important
Personal decision	○	○	○	○
Advice from family or friends	○	○	○	○
Tradition	○	○	○	○
Health deterioration	○	○	○	○
Pharmacist's recommendation	○	○	○	○
Doctor, dietitian's recommendation	○	○	○	○
Fashion	○	○	○	○

21. Where do you get information about bee products?

	Least	Little	Fairly	Much	Most
Family, relatives or friends	○	○	○	○	○
Websites	○	○	○	○	○
Popular books or magazines	○	○	○	○	○
Retailers	○	○	○	○	○
Social media	○	○	○	○	○
Television, radio	○	○	○	○	○
Pharmacists	○	○	○	○	○
Doctors, nutritionists	○	○	○	○	○
Scientific publications	○	○	○	○	○

22. Where do you buy bee products?

	Never	Most rarely	Rarely	Fairly	Often	Most often
In pharmacies	○	○	○	○	○	○
In shopping centers	○	○	○	○	○	○
In specialized stores	○	○	○	○	○	○
On the Internet	○	○	○	○	○	○
In the market	○	○	○	○	○	○
At beekeepers	○	○	○	○	○	○
At friends, relatives or acquaintances	○	○	○	○	○	○
Elsewhere	○	○	○	○	○	○

23. When purchasing honey or other bee products, how important is it to you that it be a Lithuanian product?

- ○ Very important
- ○ Not very important
- ○ Not important at all

24. How important is the type of honey according to its botanical origin to you?
Flower or nectar honey is polyfloral (multi-species) and monofloral (linden, acacia, clover, buckwheat, rapeseed, dandelion, heather, raspberry, goldenrod, etc.); leaf honey is honeydew.

- ○ Very important
- ○ It is important that it is nectar honey, not honeydew
- ○ Not very important
- ○ Not important at all

25. If you have chosen "Very important", what do you prioritize first?

- ○ Linden honey
- ○ Acacia honey
- ○ Clover honey
- ○ Buckwheat honey
- ○ Rapeseed honey
- ○ Dandelion honey
- ○ Heather honey
- ○ Raspberry honey
- ○ Goldenrod honey
- ○ Other:____

26. List what side effects of consuming bee products are you aware of?

____

27. Do you agree with the statement that bee products should not be used therapeutically in cases where the chemical justification is unknown?

- ○ Yes
- ○ No

28. What factors do you think determine the composition of bee products (amount and variety of biologically active substances)?

	Don‘t know	Has no influence	May have an influence	Has an influence
Soil properties	○	○	○	○
Seasonal meteorological conditions	○	○	○	○
Bee wintering conditions	○	○	○	○
Product collection time (in relation to the season)	○	○	○	○
Plant species	○	○	○	○

29. Why do you think it is difficult or impossible to standardize bee products?

- ☐ Bee products are usually contaminated with various impurities
- ☐ There is no standardization reference
- ☐ Many bee products are traditional (therapeutic) remedies for which standardization is not necessary
- ☐ Other: ____

Self-assessment of health status and nutritional knowledge:

30. How do you assess your health status?

- ○ Very bad
- ○ Bad
- ○ Fair
- ○ Good
- ○ Very good

31. How do you rate your level of nutritional knowledge?

- ○ Very low
- ○ Low
- ○ Fair
- ○ High
- ○ Very high
